# Quantitative analysis of renal arterial variations affecting the eligibility of catheter-based renal denervation using multi-detector computed tomography angiography

**DOI:** 10.1038/s41598-020-76812-w

**Published:** 2020-11-12

**Authors:** Won Hoon Song, Jinhwan Baik, Eue-Keun Choi, Hae-Young Lee, Hyeon Hoe Kim, Sung-Min Park, Chang Wook Jeong

**Affiliations:** 1 Department of Urology, Seoul National University Hospital, 101, Daehak-ro, Jongno-gu, Seoul, 03080 Republic of Korea; 2 Department of Urology, Pusan National University Yangsan Hospital, Yangsan, Republic of Korea; 3 Department of Creative IT Engineering, Pohang University of Science and Technology (POSTECH), 77 Cheongam-Ro, Nam-Gu, Pohang, Gyeongbuk 37673 Republic of Korea; 4 Department of Internal Medicine, Seoul National University Hospital, Seoul, Republic of Korea

**Keywords:** Cardiology, Urology

## Abstract

Catheter-based renal denervation (RDN) was introduced to treat resistant hypertension. However, the reduction in blood pressure after the RDN was modest. Catheter-based RDN was performed only at main renal arteries, except for accessory and branch arteries due to the diameter being too small for the catheter to approach. Here, we retrospectively analyzed the anatomy of diverse renal arteries via 64-channel multi-detector computed tomography angiograms of 314 consecutive donors who underwent living donor nephrectomy from January 2012 to July 2017. Occurrence rates of one or more accessory renal arteries in donors were 25.3% and 19.4% on the left and right sides, respectively. Early branching rates before 25 mm from the aorta to the right and left renal arteries were 13.7% and 10.5%, respectively. Overall, 63.1% and 78.3% of donors had no accessory artery bilaterally and no branched renal artery, respectively. As a result, 47.1% had only main renal arteries without an accessory artery and early-branching artery. Approximately half of the donors had multiple small renal arteries bilaterally, for which catheter-based denervation may not be suitable. Thus, preoperative computed tomography angiography requires careful attention to patient selection, and there is a need for improved methods for denervation at various renal arteries.

## Introduction

Resistant hypertension refers to hypertension with a blood pressure ≥ 140/90 mm Hg when treated with appropriate doses of three different types of hypertensive medications, one of which should be a diuretic^1,2^. According to the National Health and Nutrition Examination Survey, the prevalence of resistant hypertension in the United States was estimated as 8.9% of all adults with hypertension^3^.

Among previous methods for treating resistant hypertension, catheter-based sympathetic renal denervation (RDN) was the most studied^4^. Recent the multicenter, randomized, sham-controlled trial SPYRAL HTN-OFF MED showed a reduction of − 3.9 mmHg in systolic blood pressure compared to the control group after catheter-based RND treatment^5^. However, the reduction in BP was modest, so it raised questions as to the future role of renal denervation in clinical practice. Because sympathetic nerve fibers around the renal artery, which is responsible for blood pressure control, are distributed beyond the penetration depth of energy from the lumen of the artery, the previous catheter-based ablation method did not allow for complete denervation, and the possibility of intima injury increases when surgeons increase the energy to obtain deeper penetration^6,7^.

Most accessory and early-branching renal arteries have a diameter of < 3 mm^8^. Because small renal arteries measuring less than 3 mm are not indicated for catheter-based intraluminal denervation, these anatomical variations might affect the efficacy of denervation^6,9–11^. Previous clinical trials relied on conventional angiography to evaluate this arterial anatomy, and screening failure due to vascular anatomy was < 10%^12–14^. Meanwhile, our previous study indicated that more than 17% of patients had two or more renal arteries bilaterally that were identified intraoperatively^15^. Thus, many patients who had small renal arteries (< 3 mm diameter) could not be properly excluded from the previous clinical trials.

**Figure 1:**
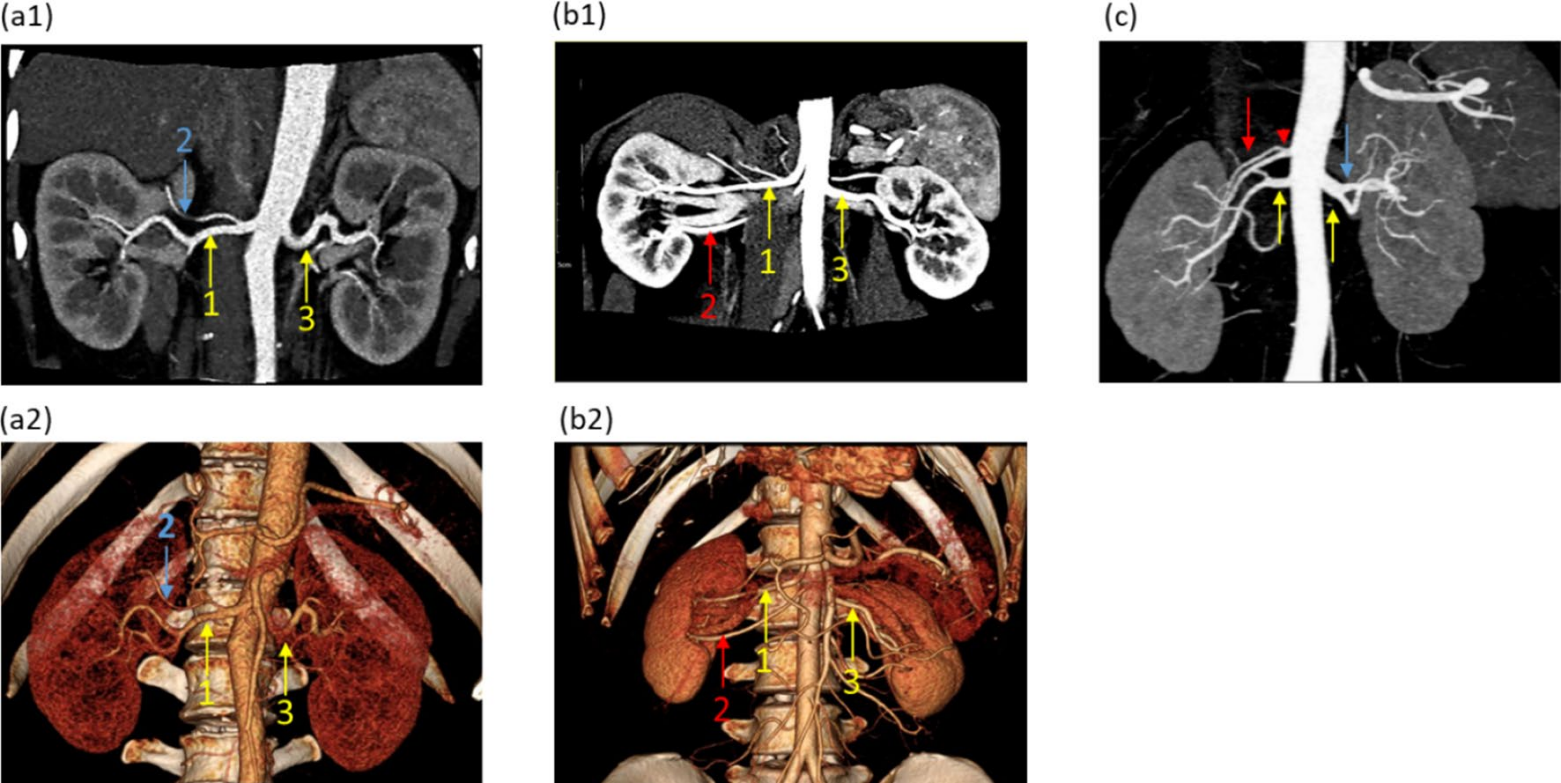
Multi-detector computed tomography angiography of the renal artery. (**a**) Main renal arteries and an early branching renal artery. (**b**) Main renal arteries and an accessory renal artery. The same numbers in (**a1**, **b1**) indicate the same arteries in (**a2**, **b2**). (**c**) A branching accessory renal artery (red arrowhead), main arteries, an early branching artery. The main, accessory, and early branching arteries are yellow, red, and blue arrows, respectively.

This study systematically evaluated consecutive patients who underwent donor nephrectomy and preoperative 64-channel multi-detector computed tomography (MDCT) angiography, which is more sensitive and accurate than conventional angiography. We aimed to estimate the frequency of small renal arteries^10,16^, investigate the anatomical background and suboptimal efficacy of catheter-based denervation through a quantitative analysis of the accessory renal artery and early-branching artery that cannot be performed using the previous catheter-based intraluminal intervention, and develop a new therapeutic strategy (Fig. 1).

## Results

The mean age of the 314 donors was 48 years (SD, 11.1). The study group included 150 (47.6%) males and 164 (52.4%) females (Table 1). There were one or more accessory renal arteries in 116 (36.9%) of the kidney donors. The incidence of an accessory renal artery was different between the right kidney (61, 19.4%) and the left kidneys, (77, 25.3%) (Table 2). Furthermore, in the right kidney, two donors had four right renal arteries. A total of 246 (78.3%) donors had no early-branching renal artery bilaterally (Table 2). Consequently, 148 (47.1%) donors had only main arteries bilaterally without accessory and early-branching arteries (Table 2).

**Table 1:** Characteristics of the patients who underwent donor nephrectomy (N = 314).

Age, mean ± SD, years	48 ± 11.1
Male sex, number (%)	150 (47.6)
Body mass index, mean ± SD, kg/m^2^	23.9 ± 3.8
Hypertensive patients, number (%)	22 (7.0)
**Type of operation, number (%)**	
Hand-assisted laparoscopic nephrectomy, left	308 (98.1)
Hand-assisted laparoscopic nephrectomy, right	1 (0.3)
Open nephrectomy, left	3 (1.0)
Open nephrectomy, right	2 (0.6)

SD, standard deviation.

**Table 2:** Incidence of accessory and branching renal arteries of donors by multi-detector computed tomography angiography.

Type of artery	None (%)	One (%)	Two (%)	Three (%)	Total (%)
**Accessory renal artery**^a^					
Right side	253 (80.6)	50 (15.9)	9 (2.9)	2 (0.6)	314 (100)
Left side	237 (74.7)	69 (21.9)	8 (2.5)	0 (0)	314 (100)
Both sides	198 (63.1)	14 (4.5)	3 (1.0)	0 (0)	
**Early branching renal artery**^b^					
Right side	271 (86.3)	43 (13.7)			314 (100)
Left side	281 (89.5)	33 (10.5)			314 (100)
Both sides	246 (78.3)	8 (2.5)			
**No accessory and early branching renal artery bilaterally**					
	148 (47.1)				

^a^Accessory artery was defined as the subartery branching directly from the aorta.

^b^Early branching artery was defined as the early branching subartery (within 2.5 cm of the aorta) from the main renal artery.

**Figure 2:**
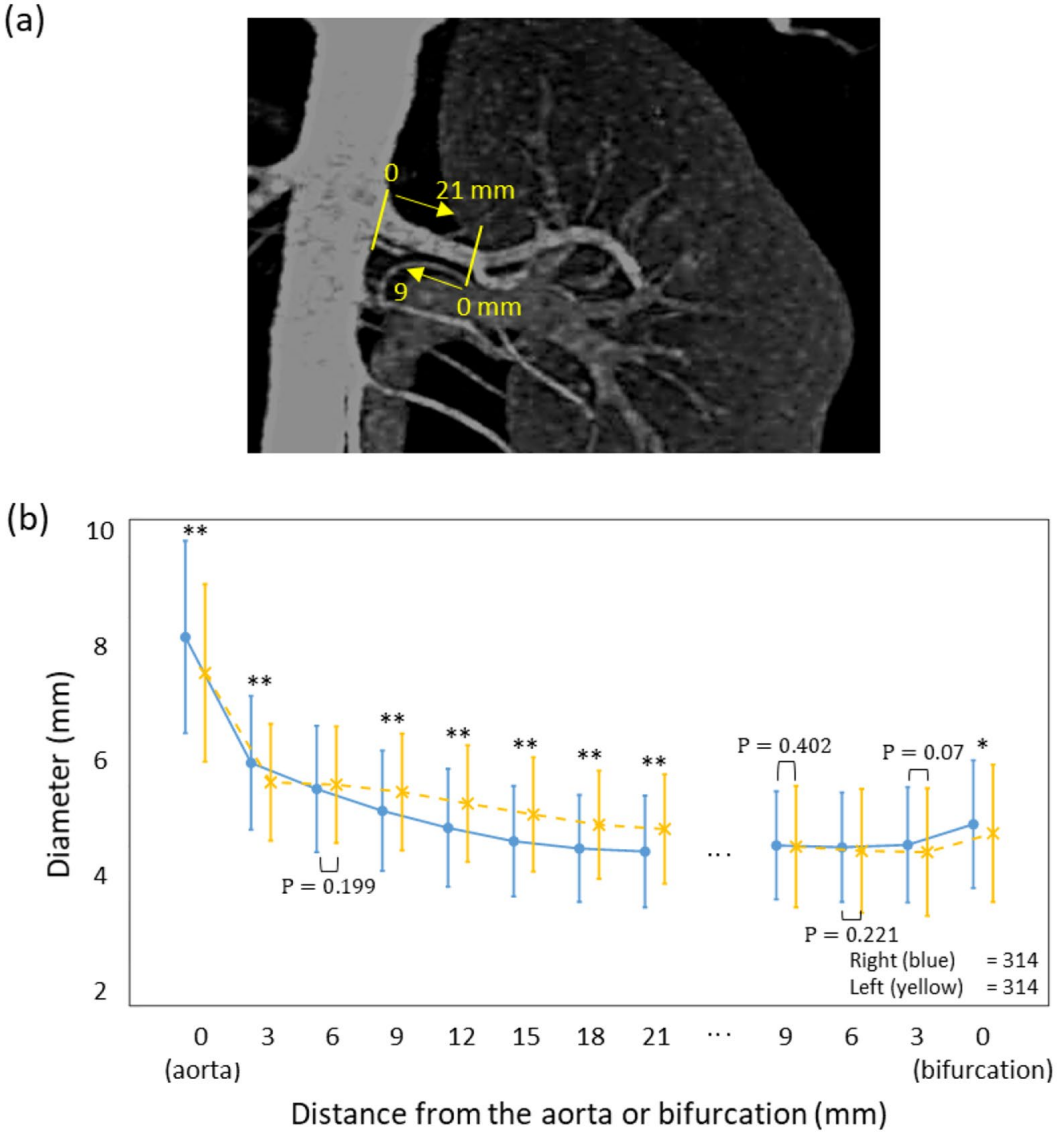
Comparison of diameters of main renal arteries by the distance from the aorta and bifurcation. (**a**) Measurement sites from the aorta are yellow and those from the bifurcation are blue. (**b**) Diameters of the main artery on the right and left sides. Points and bar present means and SD respectively. All data were met the assumptions of normality and homogeneity of variance. The diameter differences between right and left were assessed by group t-test. *p < 0.05, **p < 0.001. (Right; n = 314, Left; n = 314).

**Figure 3:**
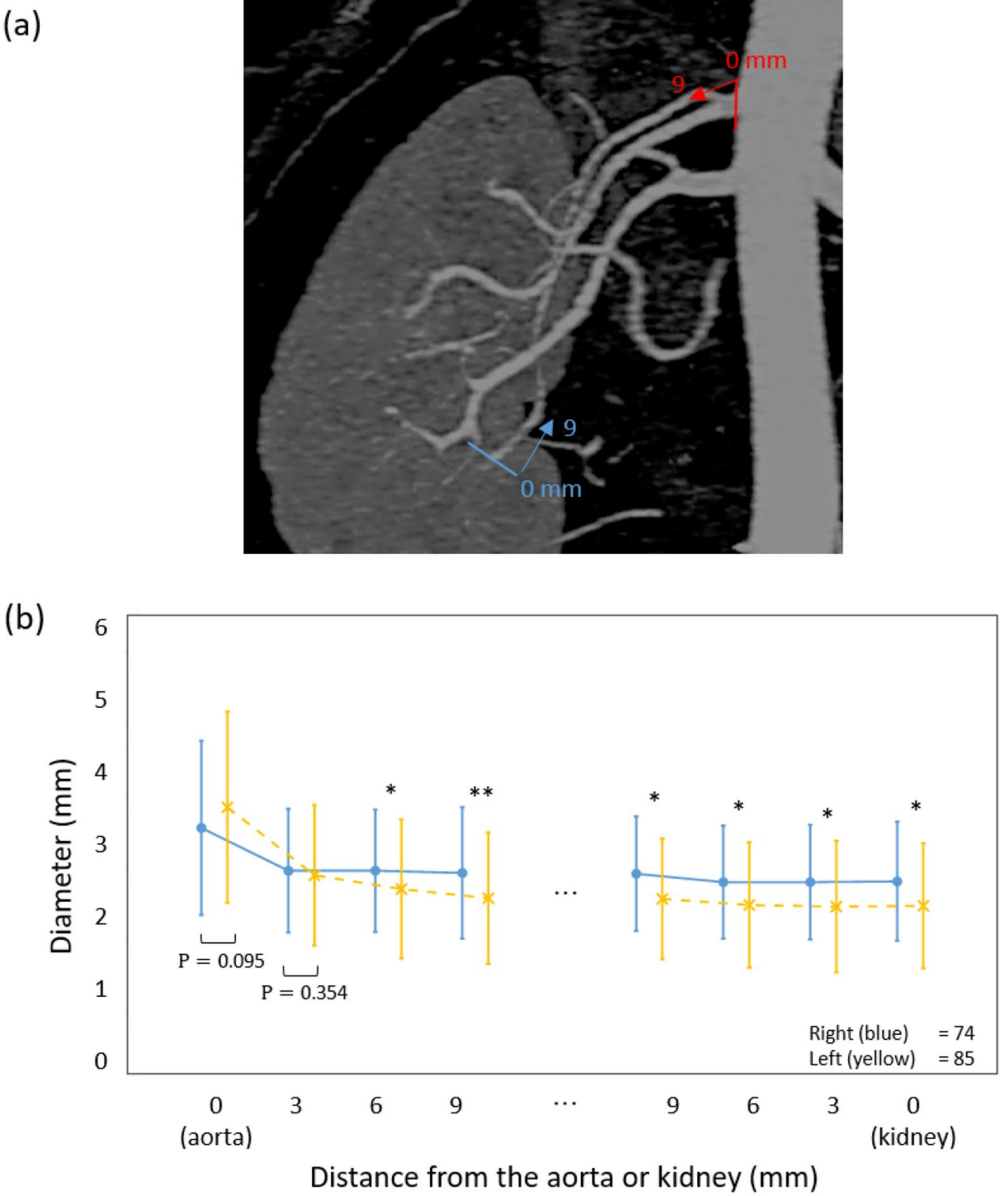
Comparison of diameters of the accessory renal arteries by distance from the aorta and kidney. (**a**) Measurement sites from the aorta are yellow and those from the kidney are blue. (**b**) Diameters of the accessory renal artery on the right and left sides. Points and bar present means and SD respectively. All data were met the assumptions of normality and homogeneity of variance. The diameter differences between right and left were assessed by group t-test. *p < 0.05, **p < 0.001. (Right; n = 74, Left; n = 85).

**Figure 4:**
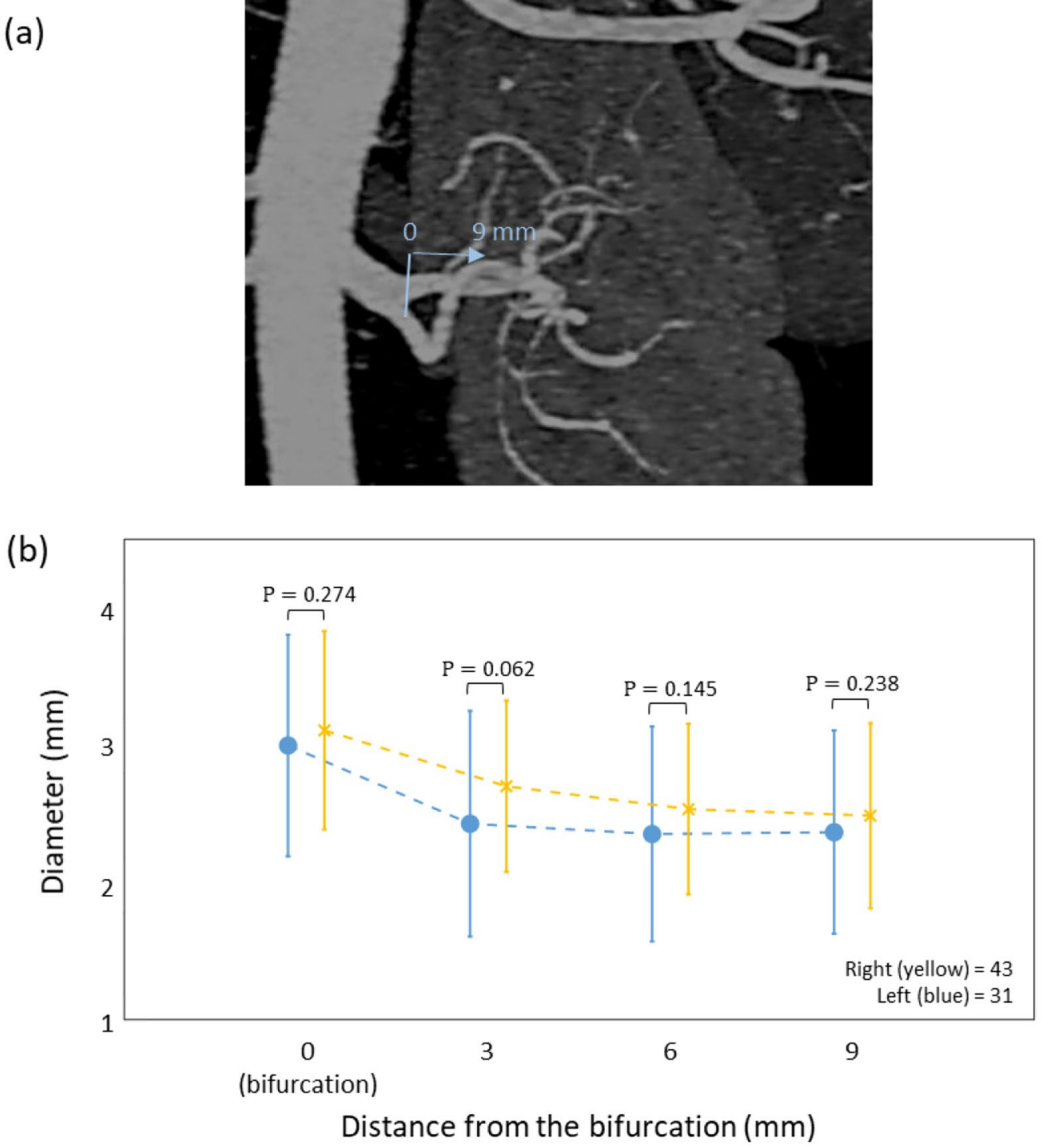
Comparison of diameters of early branching renal arteries by distance from the bifurcation. (**a**) CT image shows early branching renal artery from bifurcation to 9 mm (blue arrow). (**b**) The diameters of right (yellow) and left (blue) early branching renal arteries by distance from the bifurcation. Points and bar present means and SD respectively. All data were met the assumptions of normality and homogeneity of variance. The diameter differences between right and left were assessed by group t-test. *p < 0.05, **p < 0.001. (Right; n = 43, Left; n = 31).

We analyzed the diameters of the main renal arteries by distance from the aorta and bifurcation (Fig. 2a). Mean diameters at the bifurcation sites of the right main renal artery and left main renal artery were 4.99 mm (SD, 1.05) and 4.84 mm (SD, 1.13), respectively, and they tended to be slightly tapered toward the proximal portion at 9 mm (Fig. 2b). Mean diameters at the aortic origin of the right main renal artery and left main renal artery were 8.07 mm (SD, 1.58) and 7.48 mm (SD, 1.46), respectively, and they tended to become thinner toward the distal portion at 21 mm (Fig. 2b). In the same manner, mean diameters of accessory renal arteries by distance from the aorta to kidney (Fig. 3a) were 3.2 mm (SD, 1.14) at the right accessory renal artery and 3.49 mm (SD, 1.25) at the left accessory renal artery (Fig. 3b). From the kidney to the aorta, the diameter of the accessory arteries was 2.62 mm (SD, 0.75) and 2.29 mm (SD, 0.79), respectively. Thus, accessory arteries tended to become thinner toward the distal portion. In the early-branching renal arteries, mean diameters of the right and left renal arteries at the origin from the aorta were 2.99 mm (SD, 0.81) and 3.11 mm (SD, 0.72) respectively, and they tended to become narrower toward the distal portion at 9 mm (Fig. 4a,b). The mean length of the right main renal artery (51.56 mm; SD, 13.68) was longer than that of the left main renal artery (44.46 mm; SD, 12.40) (Table 3). The mean lengths of early branching from the aorta to the bifurcation of the right and left arteries were almost identical at 13.13 mm (SD, 6.58) and 12.69 mm (SD, 4.93), respectively (Table 3).

**Table 3:** Comparison of the length of renal arteries of donors by multi-detector computed tomography angiography.

Type of artery (N)	Total length (mm, mean ± SD)
Rt. main renal artery (314)	51.6 ± 13.7
Rt. accessory renal artery (74)	50.0 ± 13.9
Rt. early branching artery (43)	37.1 ± 12.8
Distance from the aorta and bifurcation	13.1 ± 6.6
Lt. main renal artery (314)	44.5 ± 12.4
Lt. accessory renal artery (85)	46.4 ± 13.6
Lt. early branching artery (33)	28.9 ± 7.3
Distance from the aorta and bifurcation	12.7 ± 4.9

SD, standard deviation; Rt., right; Lt., left.

**Figure 5:**
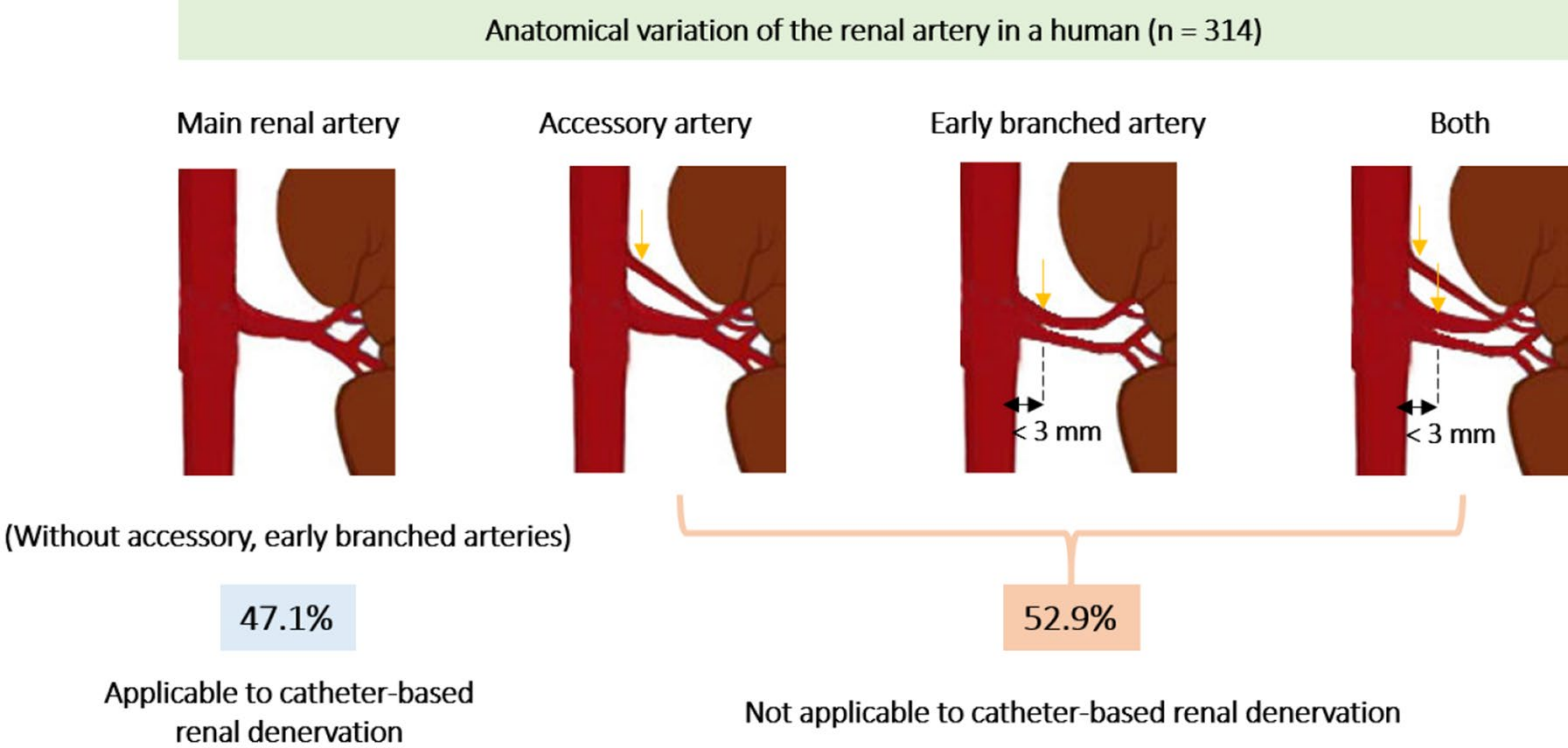
Schematic illustration of the results of this study.

To achieve optimal success in controlling BP via catheter-based RDN, patients should have a main renal artery bilaterally, without any accessory arteries and early branched arteries. Thus, only 148 (47.1%) of the 314 donors were eligible for catheter-based RDN with the recommended protocol (Table 2, Fig. 5).

## Discussion

The main findings of the study analyzing the number and diameter of renal arteries using high resolution 64-channel MDCT angiography are as follows: (1) 47.1% of kidney donors had a single renal artery bilaterally; (2) approximately 21.7% of total main renal arteries were early divided into two where the bifurcation was less than 2.5 mm away from the aorta; (3) about 36.9% of kidney donors had at least one or more accessory renal artery, and (4) the diameters of early branched main arteries and accessory renal arteries were less than 3 mm near the kidney.

### Anatomical consideration in catheter-based renal denervation

Anatomical variation can affect the protocol and the treatment effect of catheter-based RDN^6,7^. Considering severe vascular complications, catheter-based RDN may be useful only at a renal artery with a diameter of 3 mm or more^6,9–11^. However, most of the large-scale catheter-based denervation trials that evaluated arterial anatomy using conventional angiography were likely to result in poor screening for eligibility^17,18^.

In the present study, we found that 52.9% of donors had one or more accessory or early branching arteries and that the diameter of each renal artery segment varied. Whereas the diameter of the main renal artery was greater than 3 mm and was appropriate for conducting catheter-based RDN, the early branched main renal artery showed a diameter of less than 3 mm after branching. Furthermore, the diameter of all arteries was slightly tapered going from the aorta to the kidney. In our previous histological studies in humans, 31% of renal nerves were distributed 2–10 mm away from the artery and became closer to the artery as it got further from the aorta^15^. Because the penetration depth of catheter-based ablation is only about 2 mm^6^, catheter ablation should be performed at the distal renal artery for complete denervation. However, the small diameter and tapered shape of the artery would hinder complete and effective denervation. To improve denervation effectiveness, the new Symplicity Spyral multielectrode renal denervation catheter (Medtronic, Dublin, Ireland) was used to denervate branched renal arteries, rather than main renal arteries. However, it was still limited to arteries with diameters of 3–8 mm. The extended denervation to branched renal arteries was important for clinically-relevant reductions in blood pressure and enabled a more complete and circumferential ablative treatment^14^.

The above limitations occurred more clearly in accessory arteries. Thus, the current surgical protocol of catheter-based RDN was conducted only on the main renal artery^18^. However, ablation of small renal accessory arteries should be performed to improve the effect of renal denervation in the reduction of BP^19,20^. The nerves were proportionally distributed to small accessory arteries^15^.

In clinical trials, consideration of the inclusion criteria of patients with this arterial anatomy may be effective. For complete denervation, development of electrodes applicable to the diameters, numbers, and lengths of the various arteries will be of paramount importance, given these various deviations in BP reduction after RDN. These results support the fact that there are fundamental limitations to existing catheter-based intraluminal denervation and that a new strategy should be developed based on detailed quantitative information.

### High resolution 64-channel multi-detector computed tomography angiography

The anatomy of renal arteries is complicated by the branched main renal artery, multiple accessory arteries, and branched accessory arteries. Conventional angiography used for screening RDN patients is not appropriate to analyze this complex anatomy.

CT angiography is a useful clinical tool for evaluating normal vascular anatomy and diagnosing vascular disorders, especially for evaluating vasculature following renal transplantation^21–23^. MDCT is an improved imaging technology over CT angiography, however. In our previous studies with human tissue and abdominal CT review, 10% and 15% of patients had more than two renal arteries unilaterally^15^. However, the frequency of an accessory renal artery or early branching renal artery was 52.9% in this study, which used 64-channel MDCT angiography with 1-mm slices. The reason for the difference was that MDCT angiography can more accurately reflect arterial anatomical variations than CT angiography. MDCT angiography can detect small vessels measuring less than 2 mm^24^, its sensitivity to renal artery detection and location was 100%, and the correlation between surgical and CT findings was close to 95%^25,26^. Therefore, it may be advisable to use MDCT angiography before catheter-based RND and consider treatment options for patients deemed inappropriate for catheter-based RDN based on the MDCT angiography findings.

Since more than half of adults had one or more accessory arteries or early-branching arteries for which conventional catheter-based intraluminal denervation could not be performed, a catheter-based approach would not always guarantee complete denervation. Thus, our study findings suggest that anatomical determinants observed with CT angiography should be considered prior to renal denervation, and a new therapeutic strategy for complete renal denervation that can be applied to small renal arteries is strongly needed.

### Limitations

There are several limitations to this study. First, the reproducibility of the measured values was not validated by comparing the number of renal arteries on CT images with the surgical findings. Although the sensitivity of MDCT angiography is reliable for detecting the location and diameter of small renal arteries, the arterial diameter based on the CT measurement might have been slightly different from the surgical findings because of the limitations of CT resolution and marginal blurring. Second, because too many segment measurements were needed, full-range measurements of the arterial diameter from the origin of the aorta to the distal bifurcation were not performed. Lastly, CT angiography could not be used to analyze the anatomical relationship between the arteries and nerves. Although we did not analyze patients with hypertension or patients requiring renal denervation, extrapolation was appropriate because hypertension does not change renal anatomy, such as the number of arteries. In addition, because of arterial stenosis, there may be fewer patients who are eligible for catheter-based RDN in clinical practice than in this study.

## Methods

### Study design and population

Written informed consent was obtained from all patients, and this study was approved by the Institutional Review Board of Seoul National University Hospital Biomedical Research Institute (No. 2003–167-1112). The investigation conformed to the principles outlined in the Declaration of Helsinki. We retrospectively analyzed MDCT angiograms of 314 patients who underwent donor nephrectomy from January 2012 to July 2017. Only kidney donors who underwent hand-assisted laparoscopic donor nephrectomy (HAL-DN) or open donor nephrectomy in our hospital were included in this study. A small set of kidney donors who underwent other abdominal CT protocols or CT angiography in other hospitals were excluded. We routinely performed preoperative 64-channel MDCT angiography (Somatom Definition Model No. 07740777; Siemens, Munich, Germany) using the most suitable and the same protocol for evaluation of the renal artery with support from urologic radiologists, and the urologist with cooperation from a urologic radiologist processed and reconstructed the images using volumetry via Rapidia version 2.8 (Infinitt, Seoul, Korea).

### Image analysis

All axial and coronal views including the arterial phase and delay phase were analyzed, and a three-dimensional image of the volume rendering (VR) and maximum intensity projection (MIP) of the arterial phase was obtained^25^. The pre-contrast phase was sliced at 3-mm intervals, and the arterial phases were determined using an iodine contrast agent. Early arterial phases were sliced at 3 mm and 1 mm, and late arterial phases were sliced at intervals of 3 mm. The delayed arterial phase at 8 min was obtained at 3-mm intervals. Additionally, statistical iterative reconstruction algorithms, such as iterative dose reduction (iDose) of the sagittal image and iDose of the coronal image, were used at 3-mm intervals. A three-dimensional image of the VR and MIP of the arterial phase was obtained at 30-mm intervals. In particular, an iodine contrast agent was injected, and the acquisition was made at 15 s after the point where the Hounsfield Units of the descending aorta began to be imaged at 100. Three-dimensional images of the renal artery and renal vein were reconstructed using images of all phases.

Basic characteristics of the 314 donors were examined. The number, diameter, and length of the main renal artery (≥ 3 mm in diameter), accessory renal artery, and early-branching artery were evaluated. The largest artery from the aorta was defined as the main renal artery (Fig. 1a). The accessory artery was defined as the subartery branching directly from the aorta (Fig. 1b). The early-branching artery was defined as the early branching subartery (within 2.5 cm of the aorta) from the main renal artery (Fig. 1a,c)^27,28^. In addition, an early branching artery from an accessory artery was defined as a branching accessory artery (Fig. 1c). The diameter was measured at 3-mm intervals from the originating portion of the aorta to the division point in two or more consecutive branches measuring ≥ 3 mm in diameter. The diameter of the artery was defined as the length of the cross-section perpendicular to the direction of the artery running at the measurement site (Fig. 2a). The length of the main renal artery was defined as the length from the originating portion of the aorta to the division point in two or more consecutive branches measuring ≥ 3 mm in diameter (Fig. 2a)^16^.

### Statistical analysis

All numerical data are expressed as a mean and standard deviation. The chi-square test was used to compare the categorical variables between the two groups, and the independent t-test was used to compare the continuous variables between the two groups. SPSS version 23.0 (IBM Corp., Chicago, IL) was used for all statistical analyses.
